# Impact of World Health Organization (WHO) new standards on the referral pattern of infertile men for assisted reproduction

**DOI:** 10.1186/1471-2164-15-S2-P31

**Published:** 2014-04-02

**Authors:** Saad Alshahrani, Ashok Agarwal, Mourad Assidi, Adel M Abuzenadah, Damayanthi Durairajanayagam, Ahmet Ayaz, Rakesh Sharma

**Affiliations:** 1 Center for Reproductive Medicine, Cleveland Clinic, Cleveland, Ohio 44195, USA; 2 Salman Bin Abdulaziz University, College of Medicine, Saudi Arabia; 3 Center of Excellence in Genomic Medicine Research, KAU, Saudi Arabia; 4 KACST Technology Innovation Center for Personalized Medicine at King Abdulaziz University, Jeddah, Saudi Arabia; 5 MARA University of Technology, Sungai, Selangor Darul Ehsan, Malaysia

## Background

New reference values for semen parameters are significantly lower in the WHO 5th edition (2010) than in the 4th edition [1,2]. Some of the highlights of the fifth edition were: 1) subjects included in this edition had < 12 months’ time to pregnancy, 2) semen analyses results were pooled and analyzed for reference values, 3) laboratories generating the data used standardized methods for semen analysis according to WHO manual available at the time of original studies and 4) one-sided lower reference limits (5th centile) were generated and proposed as lower cut-off limits for normal values. The new reference values were aimed at providing evidence-based thresholds to assist clinicians in calculating relative fertility of the patient. The goal of our study was to examine the impact of the new WHO reference values on the referral pattern of infertile men referred for assisted reproduction (IUI, IVF/ICSI).

## Materials and methods

In this study, we examined the medical records of 362 infertile, non-azoospermic men referred to the Andrology laboratory between 2011 and 2012. Patients were divided into 2 groups; Group A: patients evaluated in 2011 by the WHO 4th edition (n = 200) [1] and Group B: patients evaluated in 2012 by the WHO 5th edition (n = 162) [2]. All patients were examined for conventional semen parameters including sperm morphology. The number of total referrals for IUI or IVF/ICSI was recorded. ART outcomes were not evaluated in this study.

## Results

A significant decline in the number of IVF/ICSI referrals was seen (71.5% vs. 53.1%; P<0.05) as well as the number of patients with abnormal sperm morphology declined significantly (76.5% vs. 30.9%; P<0.05) with the introduction of new reference values for semen analysis (WHO 5th edition). In cases referred for ART, abnormal sperm morphology was documented in 107 patients in Group A (74.8%) versus 23 (26.7%) in Group B.

**Table 1:** New WHO sperm morphology cutoff and its impact on IUI/ART referrals

Parameter	Group A (WHO 4^th^ Edition)		Group B (WHO 5^th^ Edition)	
	**n**	**%**	**n**	**%**

Number of patients	200	100	162	100

WHO Abnormal Sperm Morphology	153	76.5	50	30.9^a^

New referral for IUI/IVF/ICSI	143	71.5	86	53.1^a^

IUI	94	65.7	60	70

IVF/ ICSI	49	34.3	26	30^a^

WHO Abnormal Sperm Morphology among IUI or IVF/ICSI Referrals	107	74.8	23	26.7^a^

^a^*P* <0.05 was considered significant by chi-square test

## Conclusions

Reporting of semen parameters based on the new WHO criteria may significantly impact the management of infertile men since more men will be categorized as normal and fewer of them will be referred for ART. This will result in many men postponing additional evaluations or seeking further treatment.

## References

[B1] World Health Organization WHO laboratory manual for the examination of human semen and sperm-cervical mucus interaction 1999 Fourth Cambridge University Press

[B2] World Health Organization WHO laboratory manual for the examination and processing of human semen Fifth Geneva, Switzerland http://www.who.int/reproductivehealth/publications/infertility/9789241547789/en/

